# Stress-Induced Changes of the Skin: A Narrative Review

**DOI:** 10.7759/cureus.96285

**Published:** 2025-11-07

**Authors:** Natalia Bobok, Timur Taskesen

**Affiliations:** 1 Dermatology, INSTYTUTUM AG, Zug, CHE; 2 Dermatology, Dermaesthetik HealthCare AG, Zug, CHE

**Keywords:** chronic stress, hypothalamic–pituitary–adrenal axis, psychodermatology, sensitive skin, skin-brain axis, stress

## Abstract

Adaptation to physical and psychological stress is a fundamental biological process aimed at restoring homeostasis and maintaining health. While acute stress responses are developed for short-term survival, chronic stress has increasingly been recognized as an important trigger to a wide spectrum of systemic and dermatological disorders. Stress can exacerbate inflammatory and autoimmune dermatological conditions - such as acne, psoriasis, and atopic dermatitis. This narrative review highlights the function of the hypothalamic-pituitary-adrenal (HPA) axis, neurogenic inflammation, pigmentation disorders, and immune dysregulation of the skin as a result of the interaction between the nervous system and skin during stress. Particular attention was paid to the list of skin symptoms observed in participants exposed to prolonged stress. It also provides an overview of the limitations in modelling stress and skin interactions. By integrating molecular pathophysiology with clinical observations from a large cohort, this review presents the skin not only as a barrier protecting against adverse external influences, but as an organ actively involved in multiple systemic responses.

## Introduction and background

Stress has a multifactorial impact on the health and appearance of the skin. There is a close relationship between the skin and the brain, as both skin tissues and the nervous system originate from the ectoderm [1]. The skin and brain communicate through a network known as the skin-brain axis. This interaction leads to the production of substances that modulate the local immune, hormonal, and neural processes of the skin. These links may explain the development of concomitant skin diseases in patients with neurological and psychiatric disorders [2]. The interaction between psychological stress and skin diseases is bidirectional [3]. Psychological stress can activate the interplay among the nervous, endocrine, and immune systems, triggering the onset and progression of skin diseases. In turn, the stigma associated with these diseases increases patients’ psychological burden, thus forming a vicious cycle of stress and dermatological pathology [4]. Through its network of mechanoreceptors, chemoreceptors, nerves, muscles, and blood vessels, the skin closely interacts with the central nervous system to respond to both physical and emotional stimuli. As the body’s primary barrier against external factors and a secondary receiver of the central nervous system’s stress responses, the skin is particularly sensitive to stress [5]. Neurohormones, neuropeptides, and neurotransmitters also influence the skin. These stress-related mediators affect inflammatory, immune, and various biological processes within skin cells [6]. Catecholamines may directly affect glands, blood vessels, and smooth muscles. Such reactions ensure the body’s adaptation to extreme conditions and stressors [7]. Furthermore, the skin is not only a target for stress mediators; it is also an active participant in the stress response, particularly through a fully functional local hypothalamic-pituitary-adrenal (HPA) axis [8-10]. Investigating the pathophysiological mechanisms underlying the association between stress and the progression or development of skin diseases provides a basis for studying clinical approaches aimed at alleviating dermatological symptoms, delaying disease progression, or reducing associated risks [3,11]. This review aims to provide a general overview of skin dysfunction under stress and describe limitations of experimental modeling in studying the links between stress and the skin. The structure of dermatological symptoms observed in women exposed to prolonged stress factors is described separately.

## Review

Materials and methods

A total of 79 articles were selected for the preparation of this review. The literature search was conducted in the PubMed, Scopus, Web of Science, and Google Scholar databases using the following keywords: "skin-brain axis", "stress", "models of stress", "skin diseases", "hypothalamic-pituitary-adrenal axis", "skin barrier", "neurogenic inflammation", and combinations of these terms. Additionally, specific articles addressing the impact of stress on the skin were reviewed. The analysis primarily included original studies and review papers published from July 2003 to September 2025. During the selection process, changes in skin condition under the influence of stress factors, markers of inflammation and pigmentation, and molecular mechanisms underlying the interaction between the nervous, endocrine, and cutaneous systems were evaluated. This review is based on previously published studies and does not involve any new experiments with human participants or animals. A narrative approach was applied; therefore, the review is limited by its non-systematic methodology, which may introduce selection bias.

Dermatopathological manifestations associated with psychological stress

Relationship Between Stress and Neurogenic Skin Inflammation

Stress leads to the release of various neuroendocrine mediators as a result of HPA axis activation, inducing pathological skin immune responses [12,13]. Communication between the skin and the nervous system contributes to the development of neurogenic inflammation during stress [14]. Mast cells play a central role in this interaction, being strategically located in organs at the interface between the body and the external environment [15]. In the skin, mast cell activation can trigger inflammation, itching, and alterations in barrier function [16,17]. Numerous mast cells reside near nerve fibers, and neuroendocrine mediators released by these fibers further enhance their activity. This interaction underlies a vicious cycle of mast cell and nociceptor activation, leading to neurogenic inflammation and associated pain or itch [18,19]. Stress-induced mast cell hyperactivation has been implicated in the development and exacerbation of chronic skin diseases, such as atopic dermatitis and psoriasis. For instance, stress was shown to affect skin mast cells and their proteases in a model of atopic dermatitis-like allergic inflammation [20]. Mast cells also express receptors for HPA axis hormones on their surface, making stress-associated mast cell activation one of the key mechanisms in the exacerbation of cutaneous diseases [21].

Stress-Induced Impairment of the Skin's Barrier and Immune Function

Human skin serves as the primary protective barrier, separating the internal environment of the body from harmful external factors [22]. It enables the timely recognition of and response to a wide range of stimuli, including toxic substances, allergens, ultraviolet radiation, changes in temperature, physical damage, and the penetration of pathogenic microorganisms. The structure of the skin barrier is closely associated with the neuroendocrine and immune systems [23]. Disruption of the protective barrier and reduced antimicrobial resistance are pathophysiological manifestations that can develop under stress. Stress triggers the release of cortisol, which in turn disrupts skin barrier function. Cortisol decreases the content of lipids and structural proteins in the epidermal layers, which are critical for maintaining the barrier’s protective function. As a result, stratum corneum hydration decreases, while transepidermal water loss increases [24]. In addition to systemic effects, local cortisol release further impairs the skin barrier. Cortisone, the inactive form, may be converted to active cortisol by 11β-hydroxysteroid dehydrogenase type I (11ß-HSD1) in the skin. Elevated 11ß-HSD1 levels have been correlated with increased stratum corneum cortisol under psychological stress. Consequently, higher stratum corneum cortisol levels lead to increased basal transepidermal water loss and compromised skin integrity [25]. A prospective observational study also demonstrated a strong association between poor sleep quality, increased anxiety severity, and impaired skin barrier function [26].

Keratinocytes play a central role in maintaining skin integrity and cutaneous immune responses. They are critical for epidermal repair, contributing to both cellular proliferation and re-epithelialization following cutaneous injury. Keratinocytes secrete pro-inflammatory mediators, including cytokines and chemokines, which facilitate the activation of immune cells at the wound site, thereby initiating and sustaining the inflammatory phase of wound healing. This highlights the immune and protective functions of keratinocytes [27]. Psychological stress impairs keratinocyte differentiation. Stressed keratinocytes fail to replace cells normally produced during differentiation, which can negatively affect the skin’s protective function [28]. Regarding the skin’s antimicrobial defense, increased susceptibility to infections and impaired responses to pathogens have been associated with stress exposure. Stress also compromises the immune defense function of dermal fibroblasts in synthesizing antimicrobial peptides [29].

Stress as a Trigger for Dysregulation of Cutaneous Melanogenesis

In addition to neurogenic inflammation and the impairment of the skin’s barrier and immune functions, nervous and hormonal responses to stress have been shown to affect pigmentation processes [30]. Melanin production in melanocytes serves as a physiological and protective response, safeguarding cellular membranes and DNA from ultraviolet-induced damage [31]. Melanocytes originate from neural crest cells, which are critical structures during early embryonic development. These cells migrate through embryonic tissues, gradually reaching specific areas of the skin and hair follicles [32]. It is assumed that, at the time of their separation from the neural tube, these progenitor cells were multipotent. This characteristic not only underlies melanin synthesis but also their potential to generate other derivatives of nervous tissue, such as peripheral sensory neurons and glia, which may explain the involvement of melanocytes in responses to nervous system activation [30,33,34]. Hormonal stimulation of melanocytes via the HPA axis under stress has also been confirmed [30]. Specifically, stress induces an increase in adrenocorticotropic hormone (ACTH) levels. ACTH stimulates melanogenesis in pigment cells by increasing tyrosinase activity [35]. Elevated tyrosinase expression has been implicated in melanin overproduction, resulting in skin hyperpigmentation [36]. Stress activates tyrosinase through various signaling pathways, with the primary pathway regulating melanin synthesis being the MC1R/α-MSH (melanocortin 1 receptor/α-melanocyte-stimulating hormone) pathway [37]. MC1R is regulated by both α-MSH and ACTH [38], indicating that stress-induced ACTH release serves as a key trigger of melanogenesis.

Stress-Related Modulation of Dermatological Disease Activity and Outcomes

Some skin diseases are influenced by stress, particularly pathologies that are exacerbated under stress. These include psoriasis, vitiligo, atopic dermatitis, acne vulgaris, alopecia areata, urticaria, and lichen planus. Conditions such as pruritus, seborrheic dermatitis, and hyperhidrosis can also be triggered or worsened by stress [39-41]. In particular, several prospective cohort studies have reported a significant association between elevated stress levels and acne severity, with a strong correlation observed (p < 0.01) [42]. Beyond its effects on dermatological diseases, stress impairs the skin’s reparative capacity by reducing collagen production and the efficacy of scar treatment [43]. This pathogenesis involves a decrease in pro-inflammatory cytokine expression, leading to insufficient angiogenesis, impaired matrix repair, and delayed wound healing. Research has indicated that preoperative fear and heightened anxiety negatively affect tissue repair processes, with negative emotional states impairing normal postoperative wound healing [44]. Overall, stress represents a potential factor in the exacerbation of dermatological diseases and the deterioration of patients’ quality of life [45]. Therefore, it is important to investigate the effects of stress on skin condition, the progression of dermatological diseases, and strategies to mitigate/prevent stress-related skin deterioration.

Limitations in clinical research on stress-related dermatological responses

Experimental models play a critical role in studying the pathogenesis of stress-related disorders. The most commonly used models to investigate the effects of stress on skin diseases include the following [4]:

Chronic restraint stress (CRS): This model involves the application of a CRS protocol over a defined period. Mice are placed in ventilated restraint tubes, permitting only minimal forward and backward movement. The restraint procedure is conducted in a separate room for six hours daily over 21 consecutive days. With respect to skin effects, CRS has been shown to reduce melanogenesis [46]. CRS also affects lipid metabolism in the skin, leading to an increase in saturated fatty acids and a decrease in unsaturated fatty acids, which may contribute to inflammatory processes. Additionally, natural moisturizing factors and collagen content in the skin are reduced during CRS exposure [47].

Unpredictable chronic mild stress (UCMS): This model is used in experimental animals to study the effects of chronic, unpredictable stress. In the UCMS paradigm, rodents are exposed to a series of randomized stressors, including 14-hour food deprivation, 14-hour water deprivation, three-minute swimming, one-minute tail pinch, 0.5-hour cage shock, 24-hour soiled cage, and overnight illumination, incorporating the unpredictable nature of stressors, which is critical for modeling stress-related disorders. One stressor is applied each day for 21 consecutive days [46]. The UCMS model is highly valid, as it reproduces core symptoms of depression. A notable feature of this model is the potential reversibility of stress-induced neuropsychiatric alterations, with recovery achievable through pharmacological interventions [48]. UCMS exposure has been shown to decrease innervation in eczematous lesions in atopic dermatitis [49]. This model has also been reported to induce stress-related pigmentation disorders, with fluoxetine treatment restoring melanin synthesis, highlighting the involvement of the nervous system in dermatological alterations [50].

Chronic social defeat stress (CSDS): CSDS is an established experimental model used to study major depressive disorder. In this model, mice are subjected daily to severe physical and psychosocial stress induced by aggressive mice for three consecutive weeks. The aggressor mice are preselected and trained for consistent aggressive behavior prior to the experiment. Animals exposed to CSDS exhibit stress-related behaviors such as social avoidance, anhedonia, reduced goal-directed motivation, and anxiety-like behavior [51]. CSDS induces pathophysiological alterations, including disrupted neurotransmitter levels, impaired corticosterone synthesis, and direct dysregulation of the HPA axis function [52]. This model has also demonstrated the detrimental effects of stress on dermatological conditions. Experimental data indicate an exacerbation of atopic dermatitis, increased pruritus, and elevated disease severity index values during CSDS exposure. Moreover, CSDS has been associated with delayed recovery and worsening of atopic dermatitis-like symptoms [53,54].

These data highlight the importance of considering stress factors and their effects on skin physiology when treating dermatological diseases. However, studying the long-term effects of stress on the skin remains challenging, as most current dermatological research relies on the CRS and UCMS models [4]. These models primarily reflect short-term stress effects on the skin, whereas only prolonged exposure to chronic stress leads to cumulative physiological consequences [55]. A major limitation of chronic stress models is their poor reproducibility. Researchers often encounter difficulties in replicating key behavioral and physiological outcomes associated with chronic stress [56,57]. In addition to these challenges, inconsistencies in subject selection further complicate dermatological studies. Animal models, particularly mice, remain the gold standard for fundamental research on dermatological pathologies, as they allow researchers to reproduce disease mechanisms and evaluate potential therapeutic approaches [58]. However, comparative genomic analyses have shown only limited homology between human and mouse skin, with approximately 30% of skin-related genes shared between the two species [59]. Significant differences have been identified in genes regulating skin morphogenesis, epidermal cell proliferation, and immune functions. Rodent skin also differs anatomically from human skin, particularly in epidermal layer thickness, hair follicle density, and the presence of an additional muscle layer in mice. These distinctions limit the accuracy of extrapolating experimental findings to humans. Therefore, the development of improved models, particularly those involving human tissues, remains an important priority for studying the pathogenesis of dermatological diseases and for identifying effective therapeutic strategies [60]. Current experimental tissue models in dermatology are also constrained by the lack of vascularization and suboptimal culture methods. This underscores the need for advanced cell-based models capable of mimicking blood supply and immune cell interactions [61,62]. Furthermore, dermatological diseases are highly heterogeneous, making it difficult to obtain homogeneous patient cohorts [63]. When interpreting clinical trial data, dermatologists should not rely solely on indicators of statistical significance. It is equally important to consider participant characteristics, the magnitude of therapeutic effects, and the statistical power of the study. As study power increases, so does its ability to detect differences between groups. With the continuous advancement of modern diagnostic technologies in dermatology, assessing statistical power is important for determining both the statistical and clinical relevance of obtained results [64,65]. The following section describes the pattern of skin symptoms observed in a large cohort of women exposed to long-term (several years) psychosocial stress.

Cutaneous alterations associated with chronic stress exposure

A recent large-scale dermatological study was conducted by the company INSTYTUTUM to examine skin symptoms in women exposed to prolonged stress [66]. The study assessed the facial skin condition of 12,259 women aged 14-65 years. Participants provided facial photographs for analysis using proprietary AI-based software developed by INSTYTUTUM, which quantifies skin parameters such as redness, pore size, pigmentation, wrinkle depth, elasticity, and sebum production. The AI model, trained on dermatologically validated datasets, achieved up to 98% classification accuracy in identifying skin conditions according to predefined parameters. In addition, participants completed structured digital questionnaires evaluating subjective skin sensitivity, perceived stress levels, and environmental exposures. Data were stratified by age, symptom severity, and seasonal variation. AI-driven image processing identified the most prevalent skin conditions: (1) sensitive skin: 42% of women (mild symptoms - 40%, severe symptoms - 2%); (2) inflammatory signs: 21% (mild - 20%, severe - 1%); (3) age-related changes: 8.6% (mild - 8.2%, severe - 0.4%); (4) hyperpigmentation - 8% (mild - 7%, severe - 1%); and (5) normal skin - 20%.

**Figure 1:**
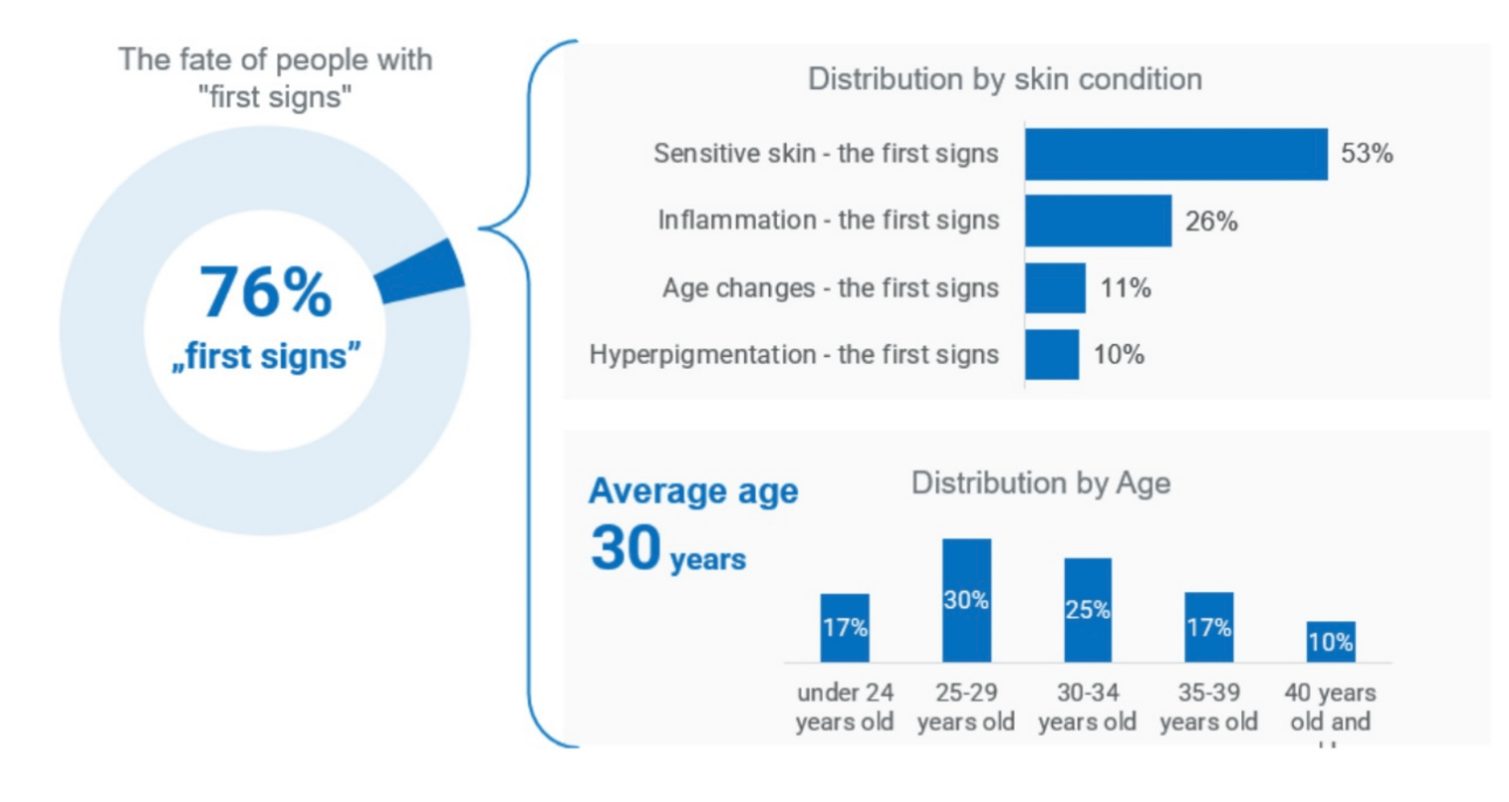
Main characteristics of patients with initial skin symptoms Adapted from Pashynska et al. [66] (an open-access journal)

The analysis revealed that sensitive skin was most commonly observed among young and middle-aged individuals: one-third of women with normal or sensitive skin belonged to the 25-29-year age group. Signs of inflammation were also more prevalent in younger participants, whereas age-related changes and hyperpigmentation were more frequently observed in older groups. In younger women, characteristic features included redness, enlarged pores, blackheads, localized rashes, couperose and vascular networks, and increased sebum production. Among participants aged 35 years and older, the prevalence of symptoms increased significantly, including pronounced nasolabial folds, uneven skin tone, reduced firmness and elasticity, loss of facial volume, and blurring of facial contours, as well as age-related changes in the neck and periocular area. Overall, early manifestations of skin changes were observed in 76% of participants. Among them, 53% primarily exhibited skin sensitivity, 26% showed inflammatory signs, 11% hyperpigmentation, and 10% age-related changes (Figure 1). Participants with severe symptoms accounted for 4% of the total sample.

**Figure 2:**
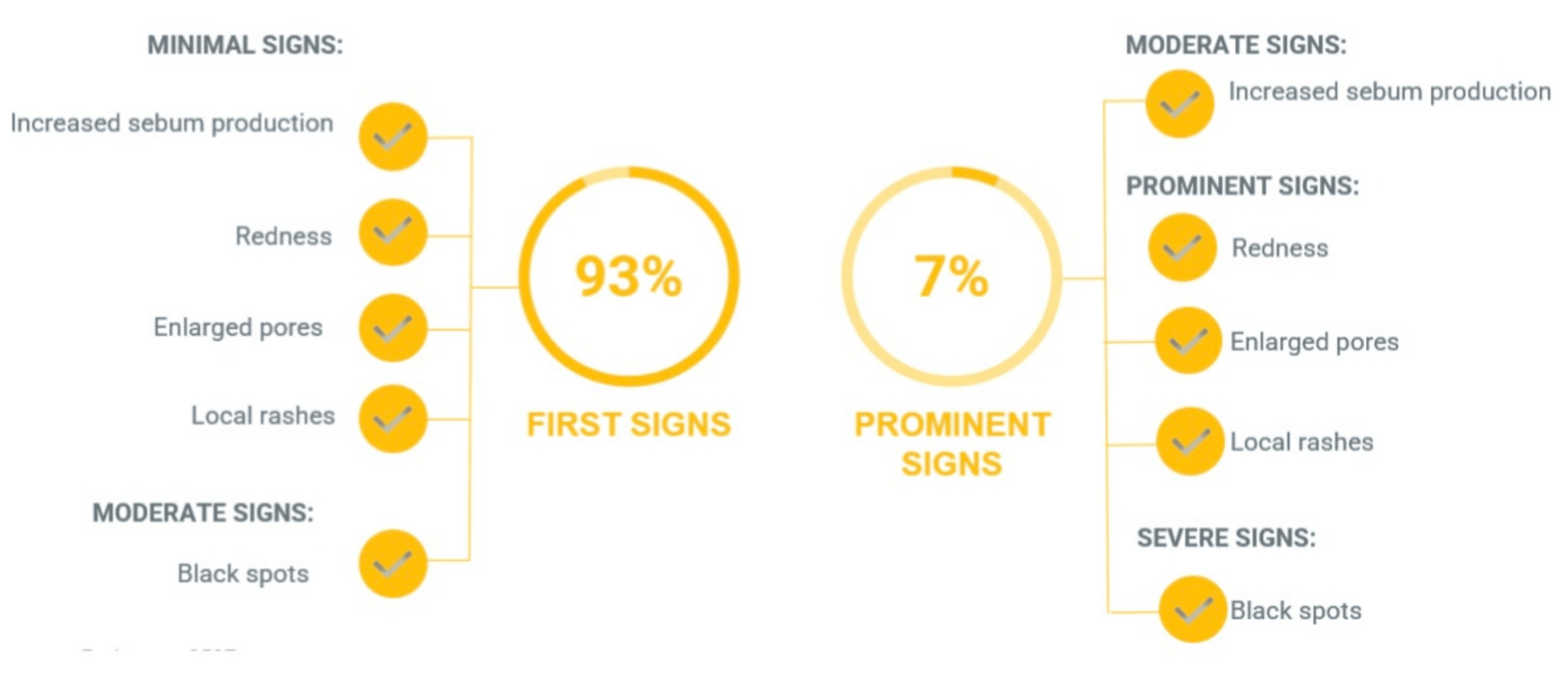
Symptom structure in the inflammatory skin type group Adapted from Pashynska et al. [66] (an open-access journal)

Among the most common symptoms observed across all age groups was redness (63%). Less frequent findings (42% each) included couperose, vascular network formation, sensations of tightness, rosacea, dryness, and peeling. Increased sebum secretion, enlarged pores, blackheads, and localized rashes were observed in 21% of participants. The least frequent manifestations (8-9%) included pronounced nasolabial folds, dull and uneven skin tone, reduced firmness and elasticity, and dark circles under the eyes. Most participants with sensitive skin (96%) initially presented with sensations of tightness, dryness, peeling, and redness. Isolated signs of increased sensitivity were also recorded in 22% of women with normal skin. Only 4% of women with sensitive skin exhibited pronounced facial changes, including couperose, vascular networks, redness, sensations of tightness, dryness, peeling, and rosacea. The highest number of treatment requests for sensitive skin with pronounced symptoms was recorded in March and April, likely due to seasonal factors such as temperature fluctuations, exacerbation of allergic diseases due to seasonal allergens, and increased sunlight exposure. Inflammatory manifestations were predominant among participants aged 14-25 and 25-35 years. Early signs of inflammation were observed in most women (93%), including mild increases in sebum production, redness, enlarged pores, and localized rashes, often accompanied by moderate blackhead formation. The remaining 7% of participants exhibited more pronounced inflammatory symptoms, characterized by moderate sebum production, redness, enlarged pores, and local rashes with visible blackheads (Figure 2).

The average age of women with inflammatory processes of the facial skin is 28 years, which confirms the prevalence of the problem of predisposition to inflammatory skin elements among younger women. The indicator of sensitive skin was recorded in 35% of women with skin inflammation. It is worth noting that, in women with pronounced inflammatory signs, this indicator reached 63%. These signs confirm the data of the relationship between the degree of skin inflammation and manifestations of sensitivity because of impaired protective functions [67]. The maximum number of visits for skin condition correction with inflammatory signs was recorded in the spring-summer period.

A noticeable increase in age-related changes was noted in older age groups, with peak graph indicators in the range of 35 years. The average age of participants with age-related changes in facial skin was 40 years. There was a stable relationship between age and degenerative-dystrophic skin processes. Most women with age-related changes (95%) had the first signs in the form of minimally reduced skin tone and elasticity, as well as moderately pronounced nasolabial folds, moderate loss of volume and blurring of facial contours, and moderate signs of age-related changes in the area around the eyes. Approximately 5% of respondents with age-related changes had pronounced signs, including increased nasolabial folds, dull color and uneven skin tone, reduced skin tone and elasticity, signs of age-related changes in the neck area, loss of volume, blurring of facial contours, and changes in the area around the eyes. The indicator “sensitive skin” was recorded in 28% of women with age-related changes, and among people with pronounced signs, its prevalence reaches 49%. Hyperpigmentation was also characteristic of older age groups. The average age of women with hyperpigmentation was 33 years. The indicator “sensitive skin” was recorded in 21% of those with hyperpigmentation, and among women with pronounced signs, its prevalence reaches 38%.

This large-scale analysis enabled the characterization of the structure of symptoms and skin signs in a large female cohort. Data from a substantial number of participants are essential for planning future randomized clinical trials, refining therapeutic approaches, and guiding the development of skincare products for diverse populations. The findings also suggest that stress represents one of the principal factors contributing to pathological skin changes, particularly in the onset and exacerbation of skin sensitivity and inflammatory conditions.

Discussions

Stress is a modifying factor in the pathogenesis of dermatological diseases, acting primarily through activation of the HPA axis. Excessive cortisol secretion and elevated neuropeptide levels cause dysregulation of the skin’s immune response, altered pigmentation, impaired barrier function, and pathological inflammation. The biochemical relationship between stress and the skin is evidenced by altered expression of inflammatory mediators, mast cell activation, and neurotransmitter imbalance, which collectively underlie the clinical manifestations of acne, psoriasis, atopic dermatitis, and other dermatoses. A large-scale study involving a substantial cohort revealed patterns of association, confirming the clinical relevance of stress-induced mechanisms in dermatology. The extensive sample enabled characterization of dermatological symptom patterns, their distribution across age groups, and potential seasonal influences on skin condition. Large-cohort studies provide a more reliable reflection of population-level trends. The high prevalence of skin sensitivity and inflammatory features among women exposed to prolonged stress indicates the role of stress as a significant trigger in the development of skin disorders. Future research should focus on elucidating the molecular and cellular mechanisms underlying stress-induced skin alterations, including the roles of cytokine profiles [68-70], epigenetic modifications [71-73], and therapeutic approaches targeting neuroendocrine imbalance in the skin [74-76]. To enhance the effectiveness of personalized therapy and improve the quality of life of patients with chronic skin diseases, it is essential to consider all potential factors that may exacerbate the course of the disease [77-79].

## Conclusions

Stress acts as an important determinant influencing the pathogenesis and clinical course of dermatological disorders. Activation of the HPA axis and excessive release of cortisol and neuropeptides lead to dysregulation of immune responses, alterations in pigmentation, impaired barrier integrity, and the development of inflammatory skin processes. The results of clinical trials have shown the role of chronic stress as a trigger for dermatological disorders such as acne, psoriasis, atopic dermatitis, and others. Data obtained from a large cohort revealed symptom patterns, including high prevalence rates of skin sensitivity and inflammation, as well as age- and season-dependent variations in clinical presentation. These findings highlight the importance of considering psychosocial stress as a significant determinant of skin health.

## References

[1] Jameson C, Boulton KA, Silove N, Nanan R, Guastella AJ (2023). Ectodermal origins of the skin-brain axis: a novel model for the developing brain, inflammation, and neurodevelopmental conditions. Mol Psychiatry.

[2] Weiglein A, Gaffal E, Albrecht A (2022). Probing the skin-brain axis: new vistas using mouse models. Int J Mol Sci.

[3] Mitchell AE (2018). Bidirectional relationships between psychological health and dermatological conditions in children. Psychol Res Behav Manag.

[4] Zhang H, Wang M, Zhao X, Wang Y, Chen X, Su J (2024). Role of stress in skin diseases: a neuroendocrine-immune interaction view. Brain Behav Immun.

[5] Vidal Yucha SE, Tamamoto KA, Kaplan DL (2019). The importance of the neuro-immuno-cutaneous system on human skin equivalent design. Cell Prolif.

[6] Moattari CR, Granstein RD (2021). Neuropeptides and neurohormones in immune, inflammatory and cellular responses to ultraviolet radiation. Acta Physiol (Oxf).

[7] Tank AW, Lee Wong D (2015). Peripheral and central effects of circulating catecholamines. Compr Physiol.

[8] Lee EY, Nam YJ, Kang S (2020). The local hypothalamic-pituitary-adrenal axis in cultured human dermal papilla cells. BMC Mol Cell Biol.

[9] Slominski A, Wortsman J, Tuckey RC, Paus R (2007). Differential expression of HPA axis homolog in the skin. Mol Cell Endocrinol.

[10] Skobowiat C, Dowdy JC, Sayre RM, Tuckey RC, Slominski A (2011). Cutaneous hypothalamic-pituitary-adrenal axis homolog: regulation by ultraviolet radiation. Am J Physiol Endocrinol Metab.

[11] Graubard R, Perez-Sanchez A, Katta R (2021). Stress and skin: an overview of mind body therapies as a treatment strategy in dermatology. Dermatol Pract Concept.

[12] Pondeljak N, Lugović-Mihić L (2020). Stress-induced interaction of skin immune cells, hormones, and neurotransmitters. Clin Ther.

[13] Lin TK, Zhong L, Santiago JL (2017). Association between stress and the HPA axis in the atopic dermatitis. Int J Mol Sci.

[14] Trier AM, Mack MR, Kim BS (2019). The neuroimmune axis in skin sensation, inflammation, and immunity. J Immunol.

[15] Ribatti D (2024). Mast cells are at the interface between the external environment and the inner organism. Front Med (Lausanne).

[16] Keith YH, Egawa G, Honda T, Kabashima K (2023). Mast cells in type 2 skin inflammation: maintenance and function. Eur J Immunol.

[17] Wang F, Yang TB, Kim BS (2020). The return of the mast cell: new roles in neuroimmune itch biology. J Invest Dermatol.

[18] Gupta K, Harvima IT (2018). Mast cell-neural interactions contribute to pain and itch. Immunol Rev.

[19] Voisin T, Chiu IM (2019). Mast cells get on your nerves in itch. Immunity.

[20] Rommel FR, Tumala S, Urban AL, Siebenhaar F, Kruse J, Gieler U, Peters EM (2024). Stress affects mast cell proteases in murine skin in a model of atopic dermatitis-like allergic inflammation. Int J Mol Sci.

[21] Woźniak E, Owczarczyk-Saczonek A, Placek W (2021). Psychological stress, mast cells, and psoriasis-is there any relationship?. Int J Mol Sci.

[22] de Szalay S, Wertz PW (2023). Protective barriers provided by the epidermis. Int J Mol Sci.

[23] Jiao Q, Zhi L, You B, Wang G, Wu N, Jia Y (2024). Skin homeostasis: mechanism and influencing factors. J Cosmet Dermatol.

[24] Maarouf M, Maarouf CL, Yosipovitch G, Shi VY (2019). The impact of stress on epidermal barrier function: an evidence-based review. Br J Dermatol.

[25] Choe SJ, Kim D, Kim EJ (2018). Psychological stress deteriorates skin barrier function by activating 11β-hydroxysteroid dehydrogenase 1 and the HPA axis. Sci Rep.

[26] Lyu F, Wu T, Bian Y, Zhu K, Xu J, Li F (2023). Stress and its impairment of skin barrier function. Int J Dermatol.

[27] Gupta RK, Wasnik P, Mondal D, Shukla D (2024). Critical role of keratinocytes in cutaneous immune responses. Explor Immunol.

[28] Cohen E, Johnson CN, Wasikowski R (2024). Significance of stress keratin expression in normal and diseased epithelia. iScience.

[29] Chan H, Li F, Dokoshi T (2025). Psychological stress increases skin infection through the action of TGFβ to suppress immune-acting fibroblasts. Sci Immunol.

[30] Ascsillán AA, Kemény LV (2024). The skin-brain axis: from UV and pigmentation to behaviour modulation. Int J Mol Sci.

[31] Solano F (2020). Photoprotection and skin pigmentation: melanin-related molecules and some other new agents obtained from natural sources. Molecules.

[32] Mort RL, Jackson IJ, Patton EE (2015). The melanocyte lineage in development and disease. Development.

[33] Todorov LG, Oonuma K, Kusakabe TG, Levine MS, Lemaire LA (2024). Neural crest lineage in the protovertebrate model Ciona. Nature.

[34] Fatieieva Y, Galimullina R, Isaev S, Klimovich A, Lemaire LA, Adameyko I (2025). Melanocytes and photosensory organs share a common ancestry that illuminates the origins of the neural crest. Commun Biol.

[35] Inoue K, Hosoi J, Ideta R, Ohta N, Ifuku O, Tsuchiya T (2003). Stress augmented ultraviolet-irradiation-induced pigmentation. J Invest Dermatol.

[36] Ni X, Luo X, Jiang X, Chen W, Bai R (2025). Small-molecule tyrosinase inhibitors for treatment of hyperpigmentation. Molecules.

[37] Herraiz C, Martínez-Vicente I, Maresca V (2021). The α-melanocyte-stimulating hormone/melanocortin-1 receptor interaction: a driver of pleiotropic effects beyond pigmentation. Pigment Cell Melanoma Res.

[38] Upadhyay PR, Swope VB, Starner RJ, Koikov L, Abdel-Malek ZA (2024). Journey through the spectacular landscape of melanocortin 1 receptor. Pigment Cell Melanoma Res.

[39] Goyal N, Prabhu SS (2023). Stress and common dermatological disorders: the psychophysiological dermatoses. Clin Derm Rev.

[40] Salari N, Heidarian P, Hosseinian-Far A, Babajani F, Mohammadi M (2024). Global prevalence of anxiety, depression, and stress among patients with skin diseases: a systematic review and meta-analysis. J Prev (2022).

[41] Alessandrello C, Sanfilippo S, Minciullo PL, Gangemi S (2024). An overview on atopic dermatitis, oxidative stress, and psychological stress: possible role of nutraceuticals as an additional therapeutic strategy. Int J Mol Sci.

[42] Zari S, Alrahmani D (2017). The association between stress and acne among female medical students in Jeddah, Saudi Arabia. Clin Cosmet Investig Dermatol.

[43] Mochel K, Bronte J, Kasaba M, Vempati A, Tam C, Hazany S (2025). The impact of psychological stress on wound healing: implications for neocollagenesis and scar treatment efficacy. Clin Cosmet Investig Dermatol.

[44] Basu S, Goswami AG, David LE, Mudge E (2024). Psychological stress on wound healing: a silent player in a complex background. Int J Low Extrem Wounds.

[45] Dixon LJ, Witcraft SM, McCowan NK, Brodell RT (2018). Stress and skin disease quality of life: the moderating role of anxiety sensitivity social concerns. Br J Dermatol.

[46] Pang S, Wu H, Wang Q, Cai M, Shi W, Shang J (2014). Chronic stress suppresses the expression of cutaneous hypothalamic-pituitary-adrenocortical axis elements and melanogenesis. PLoS One.

[47] Kitagawa Y, Hayakawa K, Oikawa D, Ikeda K, Ikeda M, Harada D, Furuse M (2022). Repeated restraint stress modifies fatty acid and amino acid metabolism in the mouse skin. J Vet Med Sci.

[48] Sharma S, Chawla S, Kumar P, Ahmad R, Kumar Verma P (2024). The chronic unpredictable mild stress (CUMS) paradigm: bridging the gap in depression research from bench to bedside. Brain Res.

[49] Lönndahl L, Lonne-Rahm SB, Nordlind K, Theodorsson E, El-Nour H (2010). Decreased innervation of eczematous skin in NC/Nga atopic mice during chronic mild stress. Immunopharmacol Immunotoxicol.

[50] Zhou L, Cai M, Ren Y, Wu H, Liu M, Chen H, Shang J (2018). The different roles of 5-HT1A/2A receptors in fluoxetine ameliorated pigmentation of C57BL/6 mouse skin in response to stress. J Dermatol Sci.

[51] Bordes J, Miranda L, Reinhardt M (2023). Automatically annotated motion tracking identifies a distinct social behavioral profile following chronic social defeat stress. Nat Commun.

[52] Yang J, Jia Y, Guo T (2025). Comparative analysis of HPA-axis dysregulation and dynamic molecular mechanisms in acute versus chronic social defeat stress. Int J Mol Sci.

[53] Yoshida Y, Hayakawa K, Fujishiro M (2020). Social defeat stress exacerbates atopic dermatitis through downregulation of DNA methyltransferase 1 and upregulation of C-C motif chemokine receptor 7 in skin dendritic cells. Biochem Biophys Res Commun.

[54] Zhao Q, Tominaga M, Toyama S (2024). Effects of psychological stress on spontaneous itch and mechanical alloknesis of atopic dermatitis. Acta Derm Venereol.

[55] Chu B, Marwaha K, Sanvictores T, Awosika AO, Ayers D (2024). Physiology, stress reaction. StatPearls.

[56] Markov DD, Novosadova EV (2022). Chronic unpredictable mild stress model of depression: possible sources of poor reproducibility and latent variables. Biology (Basel).

[57] Stanford SC (2020). Some reasons why preclinical studies of psychiatric disorders fail to translate: what can be rescued from the misunderstanding and misuse of animal ‘models’?. Altern Lab Anim.

[58] Wang M, Zhang J, Qiao C, Yan S, Wu G (2024). Comparative analysis of human and mouse transcriptomes during skin wound healing. Front Cell Dev Biol.

[59] Gerber PA, Buhren BA, Schrumpf H, Homey B, Zlotnik A, Hevezi P (2014). The top skin-associated genes: a comparative analysis of human and mouse skin transcriptomes. Biol Chem.

[60] Salgado G, Ng YZ, Koh LF, Goh CS, Common JE (2017). Human reconstructed skin xenografts on mice to model skin physiology. Differentiation.

[61] Rimal R, Muduli S, Desai P (2024). Vascularized 3D human skin models in the forefront of dermatological research. Adv Healthc Mater.

[62] Löwa A, Jevtić M, Gorreja F, Hedtrich S (2018). Alternatives to animal testing in basic and preclinical research of atopic dermatitis. Exp Dermatol.

[63] Chovatiya R, Silverberg JI (2022). The heterogeneity of atopic dermatitis. J Drugs Dermatol.

[64] Bhardwaj SS, Camacho F, Derrow A, Fleischer AB Jr, Feldman SR (2004). Statistical significance and clinical relevance: the importance of power in clinical trials in dermatology. Arch Dermatol.

[65] Silverberg JI (2015). Study designs in dermatology: practical applications of study designs and their statistics in dermatology. J Am Acad Dermatol.

[66] Pashynska KY (2025). [Analysis of Ukrainian women’s skin condition under the influence of psychological stress]. Ukr Med Journ.

[67] Kuang X, Lin C, Fu Y (2025). A comprehensive classification and analysis of oily sensitive facial skin: a cross-sectional study of young Chinese women. Sci Rep.

[68] Kunz-Ebrecht SR, Mohamed-Ali V, Feldman PJ, Kirschbaum C, Steptoe A (2003). Cortisol responses to mild psychological stress are inversely associated with proinflammatory cytokines. Brain Behav Immun.

[69] Iznardo H, Puig L (2022). IL-1 family cytokines in inflammatory dermatoses: pathogenetic role and potential therapeutic implications. Int J Mol Sci.

[70] Iznardo H, Puig L (2021). The interleukin-1 family cytokines in psoriasis: pathogenetic role and therapeutic perspectives. Expert Rev Clin Immunol.

[71] Haykal D, Will F, Cartier H, Dahan S (2025). Epigenetic modifications and the role of medical lasers in enhancing skin regeneration. J Cosmet Dermatol.

[72] Shibata S (2021). Chromatin dynamics and epigenetics in skin stress adaptation. J Dermatol Sci.

[73] Szabó K, Balogh F, Romhányi D (2025). Epigenetic regulatory processes involved in the establishment and maintenance of skin homeostasis—the role of microbiota. Int J Mol Sci.

[74] Bocheva G, Slominski RM, Slominski AT (2019). Neuroendocrine aspects of skin aging. Int J Mol Sci.

[75] Ramot Y, Böhm M, Paus R (2021). Translational neuroendocrinology of human skin: concepts and perspectives. Trends Mol Med.

[76] Slominski AT, Slominski RM, Raman C, Chen JY, Athar M, Elmets C (2022). Neuroendocrine signaling in the skin with a special focus on the epidermal neuropeptides. Am J Physiol Cell Physiol.

[77] Kim HO, Um JY, Kim HB (2025). Comprehensive approaches to diagnosis and treatment of sensitive skin. Ann Dermatol.

[78] Ghosh S, Behere RV, Sharma P, Sreejayan K (2013). Psychiatric evaluation in dermatology: an overview. Indian J Dermatol.

[79] Latheef EN, Hafi BN (2023). Psychological interventions in psychodermatological practice. Clin Dermatol Rev.

